# Extensive hepatic metastases from combined Epstein–Barr virus infection with acute hepatic failure in small cell lung cancer: A case report

**DOI:** 10.1097/MD.0000000000042991

**Published:** 2025-06-20

**Authors:** Xingxing Li

**Affiliations:** a Department of Medical Oncology, The First People’s Hospital of Linping District, Hangzhou, Zhejiang, China.

**Keywords:** acute hepatic failure, case report, chemotherapy, Epstein–Barr virus, small cell lung cancer

## Abstract

**Rationale:**

The management of small cell lung cancer patients with acute hepatic failure presents significant challenges due to contraindications for standard chemotherapeutic agents.

**Patient concerns:**

A 72-year-old male with a history of colorectal cancer (postoperative status) was admitted to our hospital with malaise, nausea, and jaundice. During hospitalization, he exhibited persistently elevated and rapidly rising alanine aminotransferase and bilirubin levels, accompanied by clinical deterioration.

**Diagnoses:**

Primary small cell lung cancer with extensive hepatic metastases and hyperbilirubinemia was diagnosed, along with concurrent Epstein–Barr virus infection, confirmed through postoperative histopathological examination and ancillary diagnostic studies.

**Interventions and outcomes:**

Following diagnosis, the patient underwent chemotherapy. Posttreatment laboratory monitoring demonstrated a progressive decline in both serum bilirubin levels and hepatic enzyme indices.

**Lessons:**

Exploring a balance between life-saving salvage therapy and respecting these contraindications is critical in optimizing outcomes for such high-risk oncology patients.

## 1. Introduction

Small cell lung cancer (SCLC) is highly malignant and prone to distant metastasis, with the most common sites of metastasis being mediastinal lymph nodes (75.3%), liver (31.6%), bone (23.7%), and brain (16.4%).^[1]^ The prognosis for patients with liver metastases tends to be poor, with 1-year survival rates of <20%.^[1]^ The first-line treatment for extensive-stage SCLC is an etoposide-based combination chemotherapy regimen, while abnormal liver function is a relative contraindication to chemotherapy.^[2]^ We report a case of an SCLC patient with acute liver failure combined with Epstein–Barr virus (EBV) infection who received chemotherapy with improved liver function, metastasis shrinkage, and good disease control to provide clinicians with the opportunity to explore the appropriate therapeutic measures for liver metastases with acute liver failure, to explore the balance between indications and contraindications, and to better serve clinical work.

## 2. Case report

**Figure 1.**
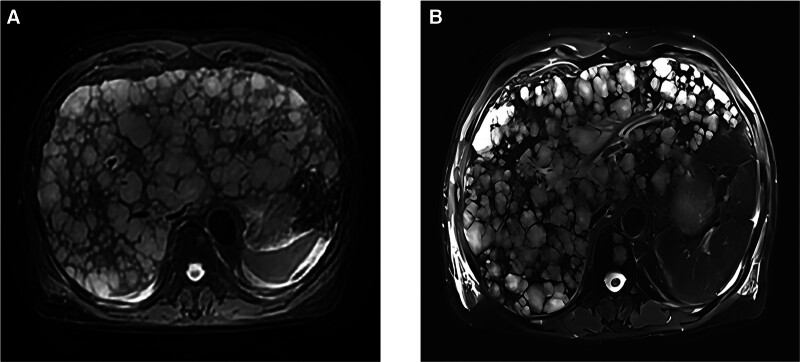
Abdominal MRI before and after treatment. (A) Patient’s abdominal MRI before chemotherapy. (B) Patient’s abdominal MRI after chemotherapy. MRI = magnetic resonance imaging.

A 72-year-old postoperative male patient with colorectal cancer presented to the hepatobiliary unit with malaise, nausea, and jaundice. Asparagine aminotransferase: 142 U/L; total bilirubin: 126.4 μmol/L. Whole abdomen enhanced computed tomography: post-radical colon surgery changes; diffuse liver metastasis was considered; liver MR: diffuse intrahepatic lesions; metastasis was considered (Fig. 1A). He was therefore referred for a medical oncology consultation.

Previous cases showed that the patient had undergone radical resection for sigmoid colon cancer 2 years ago; postoperative pathology: adenomatous carcinoma, tubular adenocarcinoma; histological grade: highly-moderately differentiated; depth of infiltration: submucosal layer: submucosal layer; margins: both margins negative; lymphovascular invasion: negative (HE); perineural invasion: negative (HE); regional lymph nodes: 0/14; peripheral lymph nodes: 0/14; perineural invasion: negative (HE); Lymph nodes: 0/14, pTNM staging (AJCC 8th edition): pT1N0M0 stage I. Regular postoperative follow-up was performed. Patients denied history of chronic diseases such as hypertension, diabetes mellitus, and chronic liver disease; history of smoking, alcohol, or drug use; and history of environmental exposure.

**Figure 2.**
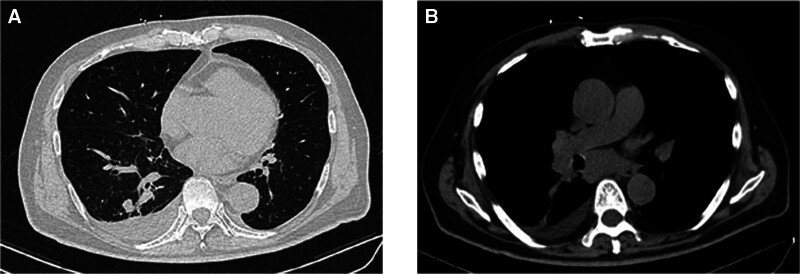
Chest CT scan before treatment. CT = computed tomography.

The patient was treated with hepatoprotective and bilirubin-lowering therapy immediately after admission, but alanine aminotransferase and bilirubin levels continued to rise rapidly, and acute liver failure (hepatocellular jaundice) was considered after multidisciplinary consultation and discussion (internal medicine, oncology, imaging, hepatic infectious disease, and hepatobiliary surgery), and the patient was treated with plasma exchange, albumin transfusion, pharmacological hepatoprotection, bilirubin lowering, nutritional support, etc. After treatment, bilirubin and aspartate transferase continued to rise, with bilirubin rising to 419.7 μmol/L and aspartate transferase rising to 388 U/L. The results of the concurrent improvement in hepatitis-related and autoimmune liver disease tests were not abnormal, and it is worth noting that the patient had positive EBV antigen expression, with an EB DNA value of 3.05E + 3 (positive). A puncture biopsy of the liver mass was performed, and the pathology revealed a (liver mass) malignant tumor, immunohistochemistry: CD56(+), Syn(+), CgA(+), INSM1(+), CDX2(-), Ki-67 (about 60%+), consistent with neuroendocrine carcinoma-small cell carcinoma. A chest computed tomography was performed: multiple enlarged lymph nodes in the mediastinum, right hilar, and supraclavicular regions bilaterally; a few nodules in the lower lobe of the right lung, newer than before (Fig. 2); and a small amount of pleural effusion on the right side. The combination of the patient’s clinical symptoms, laboratory tests, imaging studies, and the results of tissue immunohistochemistry suggested a diagnosis of primary SCLC with extensive liver metastases.

The patient was diagnosed with SCLC with extensive liver metastases combined with acute liver failure. Hepatoprotective drugs and plasma exchange therapy were ineffective, and the patient’s condition continued to deteriorate, with bilirubin and liver enzyme indices rising continuously and rapidly. After repeated communication with the family, we agreed to administer etoposide 0.1 g intravenously once daily for 5 days. The patient’s bilirubin and liver enzyme indices continued to fall after treatment (Table 1). During this period, white blood cells, red blood cells and platelets decreased, but the patient improved after symptomatic treatment with frozen plasma, recombinant human granulocyte-stimulating factor injection, recombinant human thrombopoietin injection, etc (Table 1). Three weeks later, a follow-up liver magnetic resonance imaging showed that the intrahepatic foci had shrunk compared with the previous ones. The total bilirubin was 54.9 µmol/L, aspartate aminotransferase was 23 U/L and alanine aminotransferase was 23 U/L on July 1st, 2024. Besides, white blood cells, red blood cells and platelets gradually recovered. Following that, the patient’s overall condition was still stable (Fig. 1B). Informed consent was obtained from the patient for the purpose of publication.

**Table 1:** Laboratory examination of patients.

Time	March 13, 2024	May 29, 2024	June 1, 2024	June 4, 2024	June 6, 2024	June 8, 2024	June 10, 2024	June 12, 2024	June 14, 2024	June 16, 2024	June 17, 2024	June 19, 2024	June 21, 2024	June 23, 2024	June 26, 2024	July 1, 2024
Total bilirubin (µmol/L)	39	126.5	168.8	195.4	305.8	388.6	387.7	402.1	419.7	261.8	231.8	133.7	87.9	64.1	56.4	54.9
Aspartate aminotransferase (U/L)	142	138	153	193	263	386	370	243	183	198	145	91	74	44	29	23
Alanine aminotransferase (U/L)	142	138	205	170	122	168	162	243	183	198	120	92	53	44	29	23
White blood cell count (10^9^/L)	8.1	7.9	8.6	8.3	12.9	9.2	7.3	6.3	6.9	2.9	1.2	1.1	0.8	4.3	19.1	7.4
Hemoglobin (g/L)	145	133	142	126	142	124	117	98	92	73	56	52	52	55	69	72
Absolute value of neutrophils (10^9^/L)	5.24	6.01	6.72	6.68	6.31	8	6.6	5.29	5.63	1.87	1.37	0.45	0.26	1.84	7.42	6.42
Platelet count (10^9^/L)	156	153	177	161	159	209	143	130	96	56	66	40	40	68	117	205

## 3. Discussion

This patient underwent curative surgical resection for primary colon cancer with an early-stage pathological diagnosis (pT1N0M0, stage I according to the TNM staging system). However, the patient manifested rapidly progressive, extensive hepatic metastases complicated by acute liver failure. This fulminant clinical deterioration represented an extraordinary disease acceleration, transitioning from apparent remission to terminal hepatic insufficiency within an unprecedentedly short temporal interval. Combined pathological examinations and related auxiliary tests, the patient was diagnosed as primary SCLC with extensive hepatic metastases and hyperbilirubinemia was diagnosed, along with concurrent EBV infection. It was hypothesized that EBV infection could be a contributing factor to the accelerated growth of the tumor and subsequent liver failure. The patient’s MR findings indicated diffuse hepatic infiltration, prompting the decision to administer chemotherapy, which ultimately resulted in a favorable clinical outcome.

EBV is a lymphocytophilic, double-stranded DNA virus belonging to the human herpesvirus type 4 and has been implicated in the development of tumors such as nasopharyngeal carcinoma, lymphoma, and epithelial carcinoma.^[3]^ EBV is a risk factor for prostate cancer and is associated with prostate progression.^[4]^ In another study, patients with combined HPV and EBV had higher levels of inflammatory markers (IL-17, IL-6, TNF-α, NF-κB, VEGF, ROS, and RNS), anti-regulatory regulators (Bcl-2 and survivin), and anti-nestrogenic apoptotic factors (Twist and N-cadherin).^[5]^ Viral infection may influence tumor progression by modulating cytological behavior.^[5]^ EBV genes activate cellular oncogenes or interact with host cell proteins to rapidly induce carcinogenesis.^[6]^ Potential EBV-encoded proteins and miRNAs in host cells act alone or in combination to control the cell cycle through multiple pathways. EBVs enter nasopharyngeal carcinoma cells and are subject to a period of viral lysis infection that maintains a type II latency period.^[7]^ EBV genes encode the tumor protein LMP1, which facilitates lymphangiogenesis and lymphatic metastasis of nasopharyngeal carcinoma, which is implicated in the potential for metastatic ability of nasopharyngeal carcinoma.^[7]^ EBV virus-positive prostate cancer is more likely to invade peripheral nerves.^[8]^ In light of these pathophysiological insights, the molecular stratification of EBV-associated oncogenesis emerges as a critical priority in precision oncology. However, the role between EBV and the host is a very complex relationship, and most of the current studies focus on EBV driving the host cell cycle and promoting tumor progression. Future studies will search for more precise molecular targets to provide effective solutions for the prevention and treatment of EBV-associated tumors. EBV-associated diseases are not only tumor-associated but also non-tumor-associated, such as infectious mononucleosis and hemophagic syndrome.^[9]^ Although EBV is not a hepatophagic virus, it is susceptible to liver damage manifesting as acute self-limiting hepatitis, sloughing hepatitis, and liver failure.^[10]^ While host and viral factors influence EBV-associated liver injury, the incidence of EBV-associated acute liver failure is low, approximately 0.21%, and is most common in immunocompromised patients.^[11]^ While primary EBV infection typically manifests as self-limiting hepatitis with spontaneous resolution, its progression to fulminant hepatic failure more appears in immunocompromised hosts.^[11]^ EBV may have triggered the rapid progression and metastasis of the tumor, which affected liver function, and the cancer cells destroyed the body’s immune system, which may also have caused EB-associated acute liver failure. The patient’s hepatic function showed no improvement following hepatoprotective therapy, a pattern more indicative of acute liver failure secondary to diffuse tumor infiltration than direct EBV-induced hepatic injury.

Acute liver failure is a rare complication of malignant liver disease with a high mortality rate.^[12]^ In general, acute liver failure is due to diffuse infiltration of tumor cells causing intrahepatic vascular occlusion, post-sinusoidal obstruction leading to hepatocyte death, or the release of cytokines from tumor cells leading to severe ischemic necrosis of hepatocytes.^[13]^ In this case, the patient was diagnosed with SCLC accompanied by hepatic metastases, presenting with acute liver failure secondary to severe hepatocellular dysfunction manifesting. Although standard hepatoprotective therapy was initiated, the clinical response remained suboptimal with persistent biochemical deterioration. Given the confirmed etiology of hepatic failure caused by extensive metastatic involvement coupled with the inevitable neoplastic progression without tumor control, the treatment strategy was shifted to an etoposide-based chemotherapy. This intervention achieved favorable therapeutic outcomes, evidenced by stabilization of biochemical indices and partial radiographic regression of hepatic lesions.

Metastatic SCLC has been shown to cause acute liver failure, but most patients die because of the rapid progression of the disease and the ineffectiveness of hepatoprotective drugs, which prevents them from being diagnosed and receiving appropriate chemotherapy. In a 75-year-old patient with metastatic SCLC who presented with fulminant liver abnormalities, the severity of her disease prevented her from receiving chemotherapy, and she was treated with hepatoprotective drugs, which ultimately led to her death.^[14]^ In another case of metastatic SCLC leading to fulminant liver failure, the disease was controlled after chemotherapy.^[15]^ This mode underscores that the strategic timing of interventions constitutes a pivotal determinant of disease trajectory modulation in advanced malignancies, particularly in acute-phase management for extensive-stage SCLC. Such clinical insights establish a framework for therapeutic optimization, wherein the risk-benefit calculus of early intensive regimens should be systematically evaluated while maintaining basic signs stability. However, SCLC is highly sensitive to chemotherapy and has significant short-term efficacy. However, there is a lack of long-term follow-up data in this case report, which is a limitation of the study.

## Acknowledgments

We are thankful to the patient and all the physicians and technicians who participated in this case.

## Author contributions

**Conceptualization:** Xingxing Li.

**Funding acquisition:** Xingxing Li.

**Resources:** Xingxing Li.

**Supervision:** Xingxing Li.

**Validation:** Xingxing Li.

**Writing – original draft:** Xingxing Li.
